# Investigating the effect of response autocorrelation on *n*-back analyses of serial dependence

**DOI:** 10.1167/jov.26.1.12

**Published:** 2026-01-22

**Authors:** Davide Esposito, Michele Fornaciai, Monica Gori

**Affiliations:** 1 Unit for Visually Impaired People, Istituto Italiano di Tecnologia, Genoa, Italy; 2 Life Sciences Department, University of Trieste, Trieste, Italy

**Keywords:** serial dependence, *n*-back analysis, response autocorrelation

## Abstract

Perception and decision-making in the present are not solely driven by the current inputs reaching sensory organs, but are also influenced by previous stimuli and decisions (i.e., task responses). This “serial dependence” effect is not limited to the immediately preceding stimulus or response, but it has been shown to extend several trials back in the past. However, owing to potential correlations across past responses, effects from more remote trials may be inflated, even when assessing the effect of past stimuli. In this work, we assess the potential role of response autocorrelation as a potential source of spurious results. We first show that, in serial dependence models, the effect of responses decays slowly across trials, and that such a slow decay increases the probability of observing spurious effects, even when considering past stimuli. We then provide an analytical tool to contain such spurious results. Finally, we apply our approach to a real dataset from a previous study, showing that the effect from two trials back may indeed be inflated. Our results suggest that serial dependence may be more limited in time than previously thought, and that caution is in order when assessing effects from multiple trials back in the past.

## Introduction

Our perception and decision-making are not only based on present information, but are influenced by past percepts and past decisions. The temporal context in which our perception and decisions are embedded indeed plays an important role in defining our experience and behavior. Classic examples of the influence of past stimuli are for instance the priming effect (e.g., Kristjánsson & Campana, 2010)— that is, a facilitation of current responses owing to the exposure to a previous stimulus— and *perceptual adaptation* (e.g., Kohn, 2007)— that is, a bias repulsing current percepts away from previous stimuli after a long, sustained exposure. One of these “perceptual history” effects is *perceptual serial dependence*, the bias in the judgment of a current stimulus toward previous stimuli or decisions (i.e., responses) (e.g., Cicchini, Anobile, & Burr, 2014, Fischer & Whitney, 2014). Namely, a current stimulus appears to be more similar to its preceding one than it actually is in reality, something conceptually similar to taking a weighted average of past and present (Burr & Cicchini, 2014). This results in systematic judgment errors depending on the relative difference between current and previous stimuli or decisions.

This influence is typically much subtler compared with something like perceptual adaptation, but its ubiquity in perception and decision-making suggests that it represents a generalized, fundamental mechanism. Indeed, serial dependence affects virtually every aspect of perception, starting from visual features as simple as orientation (Fischer & Whitney, 2014) or color (Barbosa & Compte, 2020), to more complex domains like numerosity (Cicchini et al., 2014; Fornaciai & Park, 2018b), and even face identity (Liberman, Fischer, & Whitney, 2014) and emotions (van der Burg, Toet, Brouwer, & van Erp, 2021). Serial dependence is not limited to vision, but also occurs in hearing (Motala, Zhang, & Alais, 2020), further supporting the generalized nature of this phenomenon.

Although the mechanisms mediating serial dependence remains largely unclear, research so far suggests that it involves both low-level and high-level brain processing stages (see for instance Manassi & Whitney, 2024). Indeed, on the one hand, serial dependence has been shown to occur from stimuli that are not task relevant (Murai & Whitney, 2021); but see Ceylan and Pascucci (2023) and Pascucci et al. (2019), and even in the absence of a task (Fornaciai & Park, 2018a), and occurs before other visual illusions based on early sensory processing (Cicchini, Benedetto, & Burr, 2021). On the other hand, serial dependence also shows some degree of abstraction from the low-level sensory properties of stimuli. For example, it works even across successive stimuli with widely different properties (Fischer et al., 2020; Fornaciai & Park, 2019a; Houborg, Kristjánsson, Tanrıkulu, & Pascucci, 2023), it depends on attention (Fischer & Whitney, 2014; Fornaciai & Park, 2018b; Fritsche & de Lange, 2019), and it is often better predicted by previous decisions than by the previous stimuli per se (Pascucci et al., 2019; Schlunegger & Mast, 2023). These results suggest that serial dependence might originate from high-level, postperceptual processes, and then potentially affect earlier perceptual processes via feedback signals (Cicchini et al., 2021; Fornaciai & Park, 2019a). That said, this may not be the only mechanism at play, as there is considerable evidence that serial dependence can emerge even without invoking feedback mechanisms propagating to early sensory cortices (Barbosa & Compte, 2020), although local recurrent interactions within cortical circuits remain possible.

Another interesting aspect of serial dependence is that it does not seem to be limited to the immediately preceding stimulus or decision, but it appears to extend further back in time to several trials in the past, especially in reproduction tasks whereby a single stimulus is presented in each trial. According to Fischer and Whitney (2014), serial dependence could thus be defined as a *continuity field*, that is, a spatiotemporally extended field determining the weight that previous stimuli or decisions exert on the perception or judgment of current stimuli (see also Manassi & Whitney, 2024 for a review). In line with this idea, studies showed that several trials back in the past can contribute to the bias in the judgment of the current stimulus (Fischer & Whitney, 2014; Morimoto & Makioka, 2022). Interestingly, using past decisions seems to more easily show effects at trials beyond the immediately preceding one (i.e., two trials back in the past, or “2-back”), while the effect of the stimuli is often confined to the most recent past trial (Blondé, Rni, Nsson, & Pascucci, 2023; Feigin, Baror, Bar, & Zaidel, 2021; Morimoto & Makioka, 2022). The superior predictive power of past decisions as opposed to past stimuli has been interpreted as serial dependence being a decisional, rather than perceptual, phenomenon (Pascucci et al., 2019), with postperceptual processes accounted for by the responses given in reproduction tasks, contributing to the serial dependence effect more than early-stage processes, accounted for by the stimuli delivered.

Considering these results, the analysis of past responses could be considered as a more accurate way to assess serial dependence in reproduction tasks (Sadil, Cowell, & Huber, 2023), and to reveal effects from trials further back in the past (Morimoto & Makioka, 2022). However, responses across different trials may not be as independent as the stimuli presented typically are, and any correlation between responses may inflate serial dependence effects across multiple past trials. This possibility is not limited to the analysis of past responses, but can potentially introduce spurious effects, even when only past stimuli are considered in the analysis. Specifically, even one single response-back autoregression can lead to a slowly decaying response of the system that can propagate beyond that sample. Such slowly decaying response can lead to the identification of relationships between current errors and past stimuli that in fact do not exist. The possibility of spurious results thus poses concerns regarding the validity of the “*n*-back” analyses conducted to date, especially in cases where multiple past trials are assessed (Blondé et al., 2023; Feigin et al., 2021; Fischer & Whitney, 2014; Morimoto & Makioka, 2022; Pascucci et al., 2019; Zhang & Alais, 2020). Assessing the validity of such analytical approaches is particularly important to reach a more accurate understanding of how serial dependence works. Indeed, the interpretation of properties like the effect of past responses and the temporal span of serial dependence have played a central role in theoretical frameworks addressing the nature of the serial dependence effect (i.e., perceptual vs. postperceptual) (Pascucci et al., 2019), as well as in defining the properties of its underlying mechanisms, like for instance the temporal span of the continuity field (Fischer & Whitney, 2014; Manassi & Whitney, 2024). In this work, we show how 1-response-back serial dependence effects can lead to spurious *n*-stimulus-back effects via simulations. Additionally, we propose a method to contain the probability of identifying spurious associations based on the removal of the 1-back response from the current error. Such a technique is finally applied to the open dataset from the seminal study of Fisher and Whitney (2014) to investigate the reliability of the 2-back effect reported in that article.

## Systems impulse response

We first analyzed the impulse response, that is, the propagation in time of a one-sample perturbation of the input to a system, in two systems similar to those used to model serial dependence effects (Fischer & Whitney, 2014; Pascucci et al., 2019), to show how they change behavior depending on which parameter, the stimulus or the response, is fed back as input.

### Methods

We compared the impulse response of two systems: one using the previous stimulus as predictor and one using the previous response. The two model formulas are the same used by Pascucci et al. (2019), with the only difference that the current error term *e*[*n*] is instead written as the difference between current response and current stimulus, *e*[*n*] = *y*[*n*] − *x*[*n*]. The two models’ formulas are the following:

(1) $$\begin{array}{c} \Delta S:\;y\left[n\right]=\;DoG\left(x\left[n-1\right]-x\left[n\right]\right)+x\left[n\right],\; \end{array}$$

(2) $$\begin{array}{c} \Delta R:\;y\left[n\right]=\;DoG\left(y\left[n-1\right]-x\left[n\right]\right)+x\left[n\right],\; \end{array}$$

where $DoG=xawce^{-{\left(wx\right)}^{2}}$, $c=\frac{\sqrt{2}}{e^{-0.5}},\;$and the impulse responses are computed for a grid of values of the parameters *a* and *w*, with *a* going from 0.01 to 0.1 and *w* going from 0.25 to 5.00. These are reasonable ranges taken from literature, like experiment 1b of the seminal work of Fisher and Whitney (2014) (*a* = 4.8,  *w* = 0.03).

### Results

**Figure 1.**
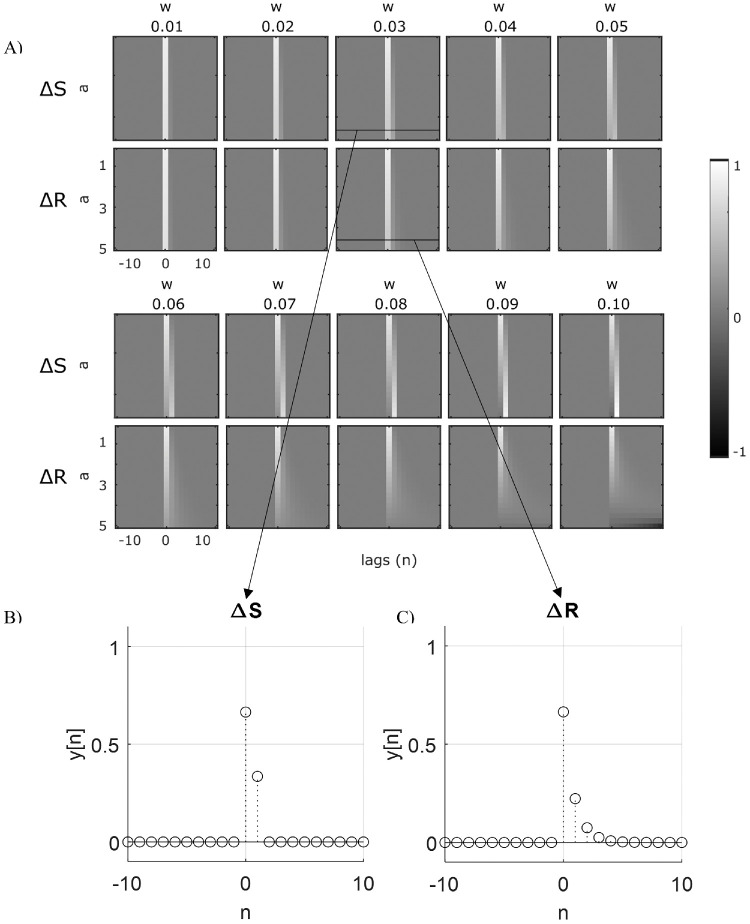
Time-course of the impulse response for various parameterizations of the generator systems (**A**). Zoom-in of the impulse response with the previous stimulus fed back, ∆S (**B**) and with the previous response fed back, ∆R (**C**) for parameters *a* = 4.8 and *w* = 0.03 (from Fischer and Whitney, 2014). The impulse response of ∆S systems decays to zero after two samples (trials) regardless of the parameterization, while the impulse response of ∆R systems decays more slowly at increasing values of *a* and *w*.

The two systems’ impulse responses are depicted in Figure 1 for various values of *a* and *w* (Figure 1A). As shown, although the ∆S system's impulse response (i.e., the impulse response of the system generated from the difference between the present and past stimulus) decreases to zero after one trial for every parameterization (e.g., for *a* = 4.8 and *w* = 0.03, *y*_Δ*S*_[0] = 0.66,  *y*_Δ*S*_[1] = 0.34,  *y*_Δ*S*_[2] = 0,  *y*_Δ*S*_[3] = 0) (Figure 1B), the ∆R system's impulse response (i.e., the impulse response of the system generated from the difference between the current stimulus and previous response (see Pascucci et al., 2019) decays more and more slowly as the *a* and *w* parameter values increase (e.g., for *a* = 4.8 and *w* = 0.03, *y*_Δ*R*_[0] = 0.66,  *y*_Δ*R*_[1] = 0.22,  *y*_Δ*R*_[2] = 0.07,  *y*_Δ*R*_[3] = 0.03) (Figure 1C), eventually introducing spurious relationships between the current error and stimuli past 1 trial back.

## Spurious associations assessment

After showing the time-course of the impulse response function of stimuli and responses, we estimated the odds of finding significant relationships between the current error and the relative distance between stimuli delivered n trials back and current stimuli, that is the typical *n*-back analysis performed to date.

### Methods

To estimate the odds of spurious *n*-back relationships, we used Monte Carlo simulations with generative models whereby the *n*-back relationship is absent and only the 1-back relationship is present. In this context, the two systems described in Equations 1 and 2 were modified by introducing a random component, ε, sampled from a normal distribution (ɛ∼N(0,1)). Therefore, the generative models are described by the following equations:

(3) $$\begin{array}{c} \Delta S:\;y\left[n\right]=DoG\left(x\left[n-1\right]-x\left[n\right]\right)+x\left[n\right]+ɛ,\; \end{array}$$

(4) $$\begin{array}{c} \Delta R:\;y\left[n\right]=\;DoG\left(y\left[n-1\right]-x\left[n\right]\right)+x\left[n\right]+ɛ,\; \end{array}$$

where $DoG=xawce^{-{\left(wx\right)}^{2}}$, $c=\frac{\sqrt{2}}{e^{-0.5}},\;\;\;$*a* going from 0.01 to 0.1 and *w* going from 0.25 to 5.00, as for the impulse response simulation. Therefore, the response term becomes a random variable *y*[*n*] ∼ *N*(*DoG*(Δ) + *x*[*n*], 1), as well as the response error term *e*[*n*] = *y*[*n*] − *x*[*n*], *e*[*n*] ∼ *N*(*DoG*(Δ), 1), with Δ being the difference between previous stimulus or response and current stimulus. Each iteration of the Monte Carlo simulation generated 1,000 trials-long signals randomly sampled from the range [0,180], as in experiment 1 of Fisher and Whitney, 2014. The Monte Carlo simulation consisted of 1,000 iterations. To estimate the odds of finding spurious significant relationships between current errors and *n*-back relative stimuli, the response error was fitted to the *n*-back relative stimulus distance (*x*[*n* − *N*] − *x*[*n*]) at each iteration using the following formula:

(5) $$\begin{array}{c} \hat{e}\left[n\right]∼DoG\left(x\left[n-N\right]-x\left[n\right];a,\;w\right).\; \end{array}$$

The fitting was performed by letting *a* as a free parameter and constraining *w* to the interval [0, 1], and it provided coefficients estimates as well as confidence intervals (CIs). The probability of spurious significance was computed as the frequency of iterations whose *a* parameter CI did not include 0. The probability of spurious significances was computed for both the Δ*S* generator and the Δ*R* generator. Finally, the ratio of the odds of spurious significance with Δ*R* generator to the odds of spurious significance with Δ*S* generator was computed to assess whether the 1-back response feedback is a risk factor for the identification of spurious *n*-back significances compared with the 1-back stimulus feedback. The analysis were conducted for *n* values going from 2 to 5. The results of the 2-back analysis are reported in detail in the Spurious associations containment section, and the results of the 3- to 5-back analyses are reported in the Supplementary Material.

### Results

**Figure 2.**
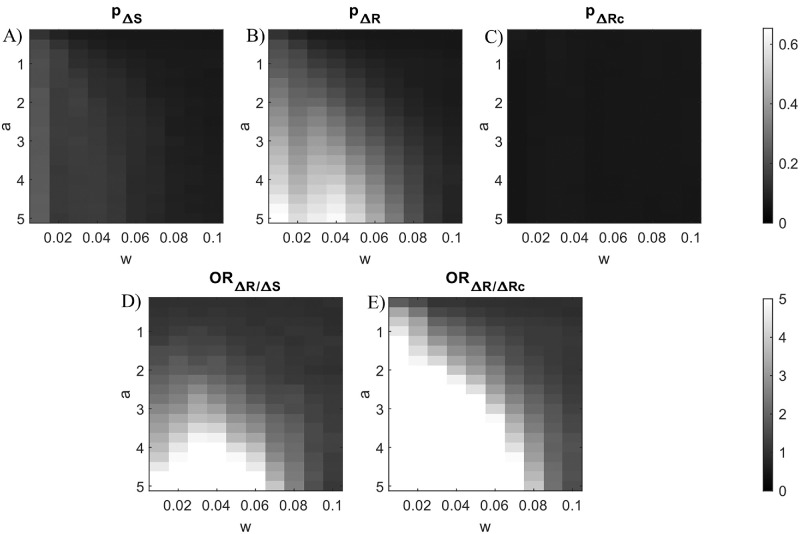
(**A**–**C**) Probabilities of spurious associations between current error and 2-back relative stimulus for different values of a and w coefficients in the 1-back generative models (Equations 3 and 4). (**A**) Probabilities for the ∆S generator model. (**B**) Probabilities for the ∆R generator model. (**C**) Probabilities for the ∆R generator model after controlling for the 1-back response effect. (**D**) Odds ratios of spurious associations for the ∆R generator model against those for the ∆S generator model. (**E**) Odds ratios of spurious associations for the ∆R generator model before against after controlling for the 1-back response effect.

The results of the Monte Carlo simulation for the 2-back analysis with various values of *a* and *w* coefficients are showed in Figure 2. The results for the 3- to 5-back analyses are showed in Supplementary Figures S1, S2, and S3. The probability of identifying a spurious 2-back effect (Figure 2A, B) grows as the values of *a* increase and the values of *w* decrease for both Δ*S* (*min*(*p*_Δ*S*_) = 0.068, *a* = 0.25,  *w* = 0.1;  *max*(*p*_Δ*S*_) = 0.249, *a* = 5,  *w* = 0.01) and Δ*R* (*min*(*p*_Δ*R*_) = 0.066, *a* = 0.50,  *w* = 0.1;  *max*(*p*_Δ*R*_) = 0.653, *a* = 5,  *w* = 0.01). The odds ratio (Figure 2D) reveals that the odds of finding spurious associations are the lowest when *a* values are small and *w* values are large ($min(OR_{\frac{\Delta R}{\Delta S}})=0.925,a=0.50,\;w=0.1$), although they are the greatest when *a* values are large and *w* values are intermediate ($max(OR_{\frac{\Delta R}{\Delta S}})=9.156,a=5,\;w=0.04$). This result suggests that, using the coefficients of nonlinearity proper of visual serial dependence on orientation reproduction tasks, the odds of identifying a spurious 2-back association between previous stimulus and current error are higher in a system including a 1-back response feedback than in a system including a 1-back stimulus feedback. The same doesn't hold for the 3- to 5-back associations, where the probability of identifying spurious associations are both low but not negligible (3-back: *max*(*p*_Δ*S*_) = 0.245, *max*(*p*_Δ*R*_) = 0.291; 4-back: *max*(*p*_Δ*S*_) = 0.272, *max*(*p*_Δ*R*_) = 0.299; 5-back: *max*(*p*_Δ*S*_) = 0.269, *max*(*p*_Δ*R*_) = 0.291) and show a similar pattern and values (3-back: $min\left(OR_{\frac{\Delta R}{\Delta S}}\right)=0.904,\;max\left(OR_{\frac{\Delta R}{\Delta S}}\right)=1.307$; 4-back: $min\left(OR_{\frac{\Delta R}{\Delta S}}\right)=0.905,\;max\left(OR_{\frac{\Delta R}{\Delta S}}\right)=1.142$; and 5-back: $min\left(OR_{\frac{\Delta R}{\Delta S}}\right)=0.900,\;max\left(OR_{\frac{\Delta R}{\Delta S}}\right)=1.179$).

## Spurious associations containment

Given the evidence reported above concerning the boosting effect of spurious 2-back associations owing to the effect of the 1-back response feedback, we propose a methodology aimed at containing such an effect. Building from the mathematical concept of partial correlation, which computes the degree of association between two variables *X* and *Y* after controlling for the effect of a third variable *Z* (Parish & Guilford, 1957), we propose to use a similar approach to identify the association between current error and *n*-back relative stimulus after controlling for the effect of the 1-back relative response. To do so, we suggest to fit the 1-back relative response onto the current error (e[n]∼DoG(y[n-1]-x[n];a,w), take that model residuals *r*[*n*], and fit the *n*-back relative stimulus onto those residuals (r[n]∼DoG(x[n-N]-x[n];a,w).

### Methods

To provide evidence for the effectiveness of such an approach in containing the identification of spurious results, this study used again a Monte Carlo simulation, using the same simulation parameters reported elsewhere in this article. However, here only the Δ*R* generator model reported in Equation 4 was used. At each iteration, i) one time series is generated from the Δ*R* model; ii) the 1-back relative response is fitted onto the current error (e[n]∼DoG(y[n-1]-x[n];a,w); iii) the fit residuals *r*[*n*] are extracted; and iv) the *n*-back relative response is fitted onto the residuals of the previous fit (r[n]∼DoG(x[n-N]-x[n];a,w). As for the previous Monte Carlo simulation, the probability of spurious significance was computed as the frequency of iterations whose *a* parameter CI for the latter fit did not include 0.

### Results

The probability of identifying a spurious association between 2-back relative stimulus and current error (Figure 2C) decreases at every combination of *a* and *w* values after removing the 1-back relative response effect from the current error (*min*(*p*_Δ*Rc*_) = 0.062, *a* = 2.25,  *w* = 0.01;  *max*(*p*_Δ*Rc*_) = 0.079, *a* = 0.25,  *w* = 0.01). Consequently, the odds ratio (Figure 2E) reveals that the odds of finding spurious associations are the lowest when *a* values are small and *w* values are large (min(ORΔRΔ Rc )=0.953,a=0.50,w=0.1), while they are the highest when *a* values are large and *w* values are small (max(ORΔRΔ Rc )=28.471,a=5,w=0.01). This result suggests that the proposed method is effective in containing the probability of identifying spurious associations in systems including a 1-back response feedback, even if it does not eliminate the risk completely. The same holds for the 3- to 5-back associations, where the probability of identifying spurious associations decreases (3-back: *max*(*p*_Δ*Rc*_) = 0.084, max(ORΔRΔ Rc )=5.904; 4-back: *max*(*p*_Δ*Rc*_) = 0.086, max(ORΔRΔ Rc )=5.186; 5-back: *max*(*p*_Δ*Rc*_) = 0.086, max(ORΔRΔ Rc )=5.681).

## Effect of containment on real associations

In the previous section, we showed that the proposed methodology successfully decreased the probability of finding a spurious association between current error and 2-back stimulus in a system including a 1-back response feedback. However, one may wonder whether the proposed correction would lead to an underestimation or loss of sensitivity in detecting the effect in a system actually including the 2-back stimulus as input. Therefore, we calculated the odds of correctly estimating the contribution of the 2-back stimulus to the current response in a system with such contribution, before and after the proposed correction.

### Methods

To investigate the effect of the proposed containment methodology on real 2-back stimulus effects, this study employed a third Monte Carlo simulation using the following system:

(6) $$\begin{array}{ccc} \Delta RS:\;y\left[n\right]\;\; & = & \;DoG\left(y\left[n-1\right]-x\left[n\right]\right)\\ & & +\;DoG\left(x\left[n-2\right]-x\left[n\right]\right)\\ & & +\;x\left[n\right]+ɛ,\; \end{array}$$

with *a* going from 0.01 to 0.10 and *w* going from 0.25 to 5.00 for the 1-back autoregressive predictor, and *a* = 5.00 and *w* = 0.04 (the parameter combination with the largest $OR_{\frac{\Delta R}{\Delta S}}$) for the 2-back stimulus predictor. The effect of the containment methodology was assessed by computing the odds ratio between the odds of estimating the correct coefficient on the ΔR*S* model before the correction and the odds of estimating the correct coefficient after the correction. The probability of estimating the correct coefficient was computed as the frequency of iterations whose *a* parameter CI for the model fit included the value of 5 (the true coefficient value).

### Results

**Figure 3.**
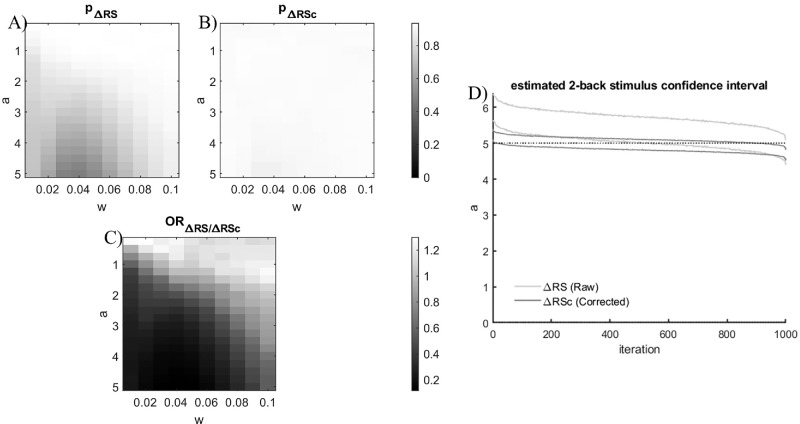
(**A**, **B**) Probabilities of identifying the correct association between current error and 2-back relative stimulus for different values of a and w coefficients in the 1-back generative model (Equation 6). (**A**) Probabilities for the ∆RS model (uncorrected). (**B**) Probabilities for the ∆RS_c_ model (corrected). (**C**) Odds ratios of identifying the correct association for the ∆RS generator model before against after the correction. (**D**) Sorted distribution of estimated confidence intervals for each iteration of the Monte-Carlo simulation for both the Δ*RS* before (light gray) and after correction (dark gray).

The results of the Monte Carlo simulation for the 2-back analysis of a system with a true 2-back stimulus association and with various values of *a* and *w* coefficients for the 1-back response association are showed in Figure 3. The probability of identifying the correct 2-back stimulus effect before the correction (Figure 3A) drops as the values of *a* increase and the value of *w* gets closer to the 2-back stimulus *w* coefficient (*min*(*p*_Δ*RS*_) = 0.472, *a* = 5,  *w* = 0.04;  *max*(*p*_Δ*RS*_) = 0.935, *a* = 0.75,  *w* = 0.04), whereas after the correction (Figure 3B) it is overall higher and less related to the parameterization ($min\left(p_{\Delta RS_{c}}\right)=0.883,a=5,\;w=0.03;\;max\left(p_{\Delta RS_{c}}\right)=0.928,a=1.25,\;w=0.02$). The odds ratio (Figure 3C) reveals that the odds of finding spurious associations are the lowest when *a* is the largest and the value of *w* gets closer to the 2-back stimulus *w* coefficient ($min(OR_{\frac{\Delta RS}{\Delta RS_{c}}})=0.118,a=5,\;w=0.04$), while they are the highest when *a* and *w* are the smallest ($max(OR_{\frac{\Delta RS}{\Delta RS_{c}}})=1.298,a=0.25,\;w=0.02$). Further inspection of the distribution of the estimates for each simulation (Figure 3D) revealed that in the raw, uncorrected models, the probability of estimating the correct coefficient value is lower because the model overestimates the effect. This result highlights that the proposed correction not only maintains the sensitivity in detecting the true 2-back stimulus effect, but it is also robust to the coefficient overestimation related to the presence of the 1-back response association.

## Dataset reanalysis

In light of the findings obtained from the simulations described, this study aimed to reanalyze the data of Fischer and Whitney's experiment 1b (Fischer & Whitney, 2014), one of the first works on perceptual serial dependence that introduced the *n*-back analysis on visual orientation reproduction.

### Methods

#### Dataset

The dataset used was retrieved from the Open Science Framework repository, published by Sadil et al. (2023). The dataset contained the data of the four participants performing the visual orientation reproduction task described in Fischer and Whitney's work (2014). This dataset was selected for two reasons. First, it comes from one of the first studies concerning serial dependence, which laid the ground for the development of such research field. Second and most important, whereas the study originally did not take into account the contribution of the response to the serial dependence effect, recently Sadil et al. reanalyzed the data identifying instead a significant 1-back contribution of the response, suggesting that the model using Δ*R* as predictor (Equation 4) is a better description of the underlying process than the model using Δ*S* (Equation 3), thus making such dataset an ideal test case for our hypothesis concerning the 2-back analysis.

#### Analysis

The data were analyzed using a method like the one described by (Pascucci et al., 2019). The analysis used nonlinear mixed effect models (NLMEM) of the form:

(7) $$\begin{array}{c} y_{ij}=b_{i}+DoG\left(x_{ij};a_{i},w\right)+{ɛ}_{ij},\; \end{array}$$

with *i* the subject index and *j* the *jth* trial, and *DoG*(*x_ij_*; *α*,*w*) given by:

(8) $$\begin{array}{c} \left\{\begin{array}{c} DoG\left(x_{ij};a_{i},\;w\right)=x_{ij}ca_{i}wx_{ij}e^{-{\left(wx_{ij}\right)}^{2}}\\ a_{i}=a_{0}+\alpha_{i}+\nu_{i}\\ b_{i}=b_{0}+\beta_{i}+\psi_{i} \end{array},\right.\; \end{array}$$

where *x_ij_* is the predictor variable, (*a_i_*, *b_i_*, *w*) are unknown parameters, with *a_i_*, ɛ R the height of the peak and valley of the curve, *w*, ɛ R the spread of the curve, *b_i_*, ɛ R a model offset (or intercept), and $c=\frac{\sqrt{2}}{e^{-0.5}}$ the normalization constant. *a_i_* and *b_i_* are formed by a group-level (*a*_0_, *b*_0_) component and an individual-level (α_*i*_, β_*i*_) component. ν_*i*_ and ψ_*i*_ are the group-level error assumed to be independent and identically distributed as $\nu_{i},∼N\left(0,\sigma_{\nu }^{2}\right)$, $\psi_{i},∼N\left(0,\sigma_{\psi }^{2}\right)$, ${ɛ}_{ij}∼N\left(0,\sigma_{ɛ}^{2}\right)$ is the residual model error, independent of ν_*i*_.

Before the analysis, the systematic biases in orientation judgements (Balikou et al., 2015) were removed from the participants errors by fitting on them a ninth degree polynomial and taking the residuals of such fits as the new participants errors (Manassi, Liberman, Kosovicheva, Zhang, & Whitney, 2018). Then, the trials whose error values were further than 3 standard deviations from the mean were deemed as outliers ; therefore, those trials and the following ones were removed from the analysis. The trials at the beginning of each experimental block were removed from the analysis as well.

To compute the association between current error (*e_ij_*) and 2-back relative stimulus orientation (*x*_*ij* − 2_ − *x_ij_* ). A first NLMEM was fitted onto the participants’ errors, using the relative orientation response as a predictor. From that model, the residuals *r_ij_* are extracted and used as response variable for a second NLMEM:

(9) $$\begin{array}{c} \left\{\begin{array}{c} e_{ij}=b_{i}^{\left(1\right)}+DoG\left(y_{ij-1}-x_{ij};a_{i}^{\left(1\right)},w^{\left(1\right)}\right)+r_{ij}\\ r_{ij}=b_{i}^{\left(2\right)}+DoG\left(x_{ij-2}-x_{ij};a_{i}^{\left(2\right)},w^{\left(2\right)}\right)+{ɛ}_{ij} \end{array}\right..\; \end{array}$$

The amplitude of the *a*_0_ coefficient was used as association strength index. The association was deemed significant if the *a*_0_ coefficient's CI did not include 0. Such NLMEM was compared with a reference NLMEM in which the 2-back relative responses were fitted directly onto the current errors:

(10) $$\begin{array}{c} e_{ij}=b_{i}^{\left(3\right)}+DoG\left(x_{ij-2}-x_{ij};a_{i}^{\left(3\right)},w^{\left(3\right)}\right)+{ɛ}_{ij}.\; \end{array}$$

To further assess the efficacy of the proposed containment method, a permutation test was performed on the amplitude of the difference between $a_{0}^{\left(3\right)}$ and $a_{0}^{\left(2\right)}$. The permutation test consisted of sampling without replacement from the participant errors, balancing for each participant, and repeating the analysis described above on the resampled data. The procedure was repeated 1,000 times to create a distribution of values in which the null hypothesis of no difference between $a_{0}^{\left(3\right)}$ and $a_{0}^{\left(2\right)}$ is true. The permutation test's *p* value was computed as the probability of having a difference $a_{0_{permutation}}^{\left(3\right)}-a_{0_{permutation}}^{\left(2\right)}$ larger than $a_{0}^{\left(3\right)}-a_{0}^{\left(2\right)}$.

All analyses were performed in MATLAB. The nonlinear mixed effects model fitting was performed with the function *nlmefit* (MaxIter = 200, TolX = 1e-4, starting parameters: *a*_0_ =2, *w* = 0.05, Approximation Type = REMLE), while the CIs for the estimated parameters were estimated using the function *nlparci*. The permutation test was performed via a custom script.

### Results

The NLMEM fitting the 2-back relative responses onto the raw current errors (Equation 10) estimated a significant 2-back serial dependence ($a_{0}^{\left(3\right)}=0.90,\;95\%\;\mathrm{CI},\;0.10\;-1.71)$. Instead, The NLMEM fitting the 2-back relative responses onto the residualized current errors (Equation 9) estimated a nonsignificant 2-back serial dependence (a0(2)=0.42,95% CI ,-1.11 to 1.96). The permutation test indicated that $a_{0}^{\left(3\right)}-a_{0}^{\left(2\right)}$ is significantly larger than zero (*p =* 0.032), supporting the hypothesis that the serial dependence effect in visual orientation reproduction involves information that is retained for one trial only. This finding is in line with previous studies that found an interfering effect of the intervening stimulus presented at one trial back (Ceylan & Pascucci, 2023; Fornaciai & Park, 2019a).

## Discussion

In the present work, we addressed the possibility that correlations across responses in consecutive trials might introduce spurious serial dependence effects, inflating the strength of the bias from stimuli presented in trials further back in the past. Indeed, responses across different trials may not be as independent as the stimuli presented, and any analyses not considering such correlation may find serial dependence effects when none exist or increase their strength. This effect is possible not only for analyses using past responses to account for serial dependence effects (Morimoto & Makioka, 2022), but also when the past stimuli are used to assess effects at different trials back in the past (Fischer & Whitney, 2014). Our results show that 1) the system impulse response corresponding to previous judgments decays slowly and persists across different trials, and that 2) such slow decay increases the probability of observing spurious serial dependence effects, even when considering the past stimuli. According to these findings, we provide an analytical tool allowing to decrease the influence of such spurious effects. Finally, by applying our approach to a real dataset (Fischer & Whitney, 2014), we show that the effect of the stimulus at two trials back in the past is indeed inflated, potentially leading to an overestimation of the temporal span of the effect (i.e., the span of the “continuity field”).

This potential spurious effect is thus very important when interpreting the extent of the continuity field. According to our results, current estimates of the temporal or trial span (i.e., 10–15 seconds, or 2–3 trials back) of serial dependence effects (e.g., Fischer & Whitney, 2014; see Manassi & Whitney, 2024 for a review) may in fact be overestimated. Although the influence of the immediately preceding stimulus is not affected by this, how long the effect spans back in the past might be more limited compared with what has been previously considered, or the effect of past stimuli might be weaker. For instance, in the case of the effects shown by Fischer and Whitney (2014), our analysis reveals that the 2-back effect mostly reflects the autocorrelation of responses, and not a genuine serial dependence effect. In our analysis, the 2-back effect is indeed not significant, suggesting an influence limited to the immediately preceding trial. Related to this finding, previous results also show that the extent of serial dependence effects is determined by a combination of both time elapsed and number of intervening stimuli (Ceylan & Pascucci, 2023; Fornaciai & Park, 2019b). This shows that serial dependence— and hence the underlying continuity field— does not only decay with time, but it is also disrupted by intervening stimuli. For instance, Fornaciai and Park have shown that even a single, neutral and irrelevant stimulus is enough to almost completely abolish serial dependence (Fornaciai & Park, 2019b). Similarly, Ceylan and Pascucci (2023) show that intervening task-relevant (“target”) stimuli also reduce serial dependence effects, although irrelevant intervening stimuli instead boost the effect of previous targets. Overall, considering the interference of intervening relevant stimuli, it is plausible that serial dependence effects might in fact be limited to the immediately preceding stimulus, with more remote effects emerging as a byproduct of response autocorrelation. An interesting prediction of our model is that the amount of autocorrelation between responses (i.e., for instance at the interindividual level) should determine the strength of effects from trials further back in the past (i.e., the strength of the 2-back effect). This notion, however, remains an open question that should be addressed by future studies.

Such inflated effects owing to response autocorrelation might also explain observations of stronger effects from 2-back responses compared with 2-back stimuli (Morimoto & Makioka, 2022). When using the responses instead of the past stimuli, effects from two trials back might be even more overestimated, leading to more prominent response-induced effects. Several studies so far have proposed that past responses provide a better index to assess serial dependence compared with previous stimuli (Pascucci et al., 2019; Sadil et al., 2023). Our results, however, suggest that caution is in order when interpreting the influence of past responses, because their autocorrelation could lead to an overestimation of effects from more remote trials— perhaps even more so than considering the past stimuli.

To control for such spurious effects, we propose a simple method: regressing out the response at 1-back from the estimation error in the current trial. This strategy is sufficient to remove, or at least reduce, spurious associations between the current estimation error and more remote stimuli owing to the response autocorrelation. The fact that using this method eliminates the 2-back effect in Fischer and Whitney's (2014) data does not, however, mean that 2-back effects do not exist at all. As discussed elsewhere in this article, the strength of more remote effects is modulated by an interaction between the passage of time, the number of stimuli, and their relevance (Ceylan & Pascucci, 2023). Paradigms with shorter intertrial intervals might show significant 2-back effects even when removing the influence of the 1-back response. In general, we believe that controlling for spurious effects owing to response autocorrelation is an important additional step for future studies, to provide a more realistic estimation of the extent of serial dependence effects. Taking this into account, another interesting goal for future studies is to more comprehensively assess the limits of the continuity field while controlling for spurious results.

This study has some limitations. First, our approach provides a mere mathematical perspective on the effect of the response autocorrelation on *n*-back analyses, focusing on isolating the contribution of the 1-back autoregression to ensure that any residual association with remote stimuli reflects real stimulus-driven effects rather than persistence of prior responses. In other words, although a response may exert influence across multiple subsequent trials owing to slow decay, our approach clarifies that this influence originates from that response rather than from earlier stimuli. However, it does not provide any means to investigate the functionality of such autocorrelation, nor its origin, being it the temporal integration of information occurring in decision-making processes or the slow decay of information in the working memory. Investigating the functional role of the slow-decaying effect of response autocorrelation and its underlying mechanism is beyond the scope of this work and dedicated investigations should be conducted to understand how and why response autocorrelation emerges, if present, in the specific task.

Second, this study does not account for long-range effects, such as those reported by Fritsche, Spaak, and de Lange (2020). In the simulation performed here, the confounding effect of the 1-back response affects mostly the 2-back stimulus, and decreases significantly, becoming relatively negligible, already at 3 trials back. Further research is, therefore. necessary to understand what mechanisms drive long-range serial dependence effects and eventually how to distinguish them from the typically investigated short-range effects.

To conclude, our findings show that, when assessing serial dependence effects at trials more remote than the immediately preceding one, and especially two trials back in the past, the bias might be inflated by response autocorrelation. This in turn inflates the apparent temporal extent of the serial dependence effect and its underlying continuity field, which might in fact be limited to the immediately preceding stimulus. We thus propose a simple yet effective method to reduce spurious results, by removing the 1-back response from the current estimation error, which indeed revealed spurious effects in our re-analysis of Fischer and Whitney's (2014) data. Overall, our results provide a new perspective on the analytical procedures involved in serial dependence research and highlight an important pitfall of the current analytical approaches that can easily be avoided to reach a better understanding of serial dependence and its underlying continuity field.

## Supplementary Material

Supplement 1
