# Supplementary material for: Investigating the effect of response autocorrelation on n-back analyses of serial dependence
Source: J Vis. 2026 Jan 22;26(1):12. doi: 10.1167/jov.26.1.12 (PMC12849821; doi:10.1167/jov.26.1.12)
Supplement: Supplement 1 [file jovi-26-1-12_s001.docx]

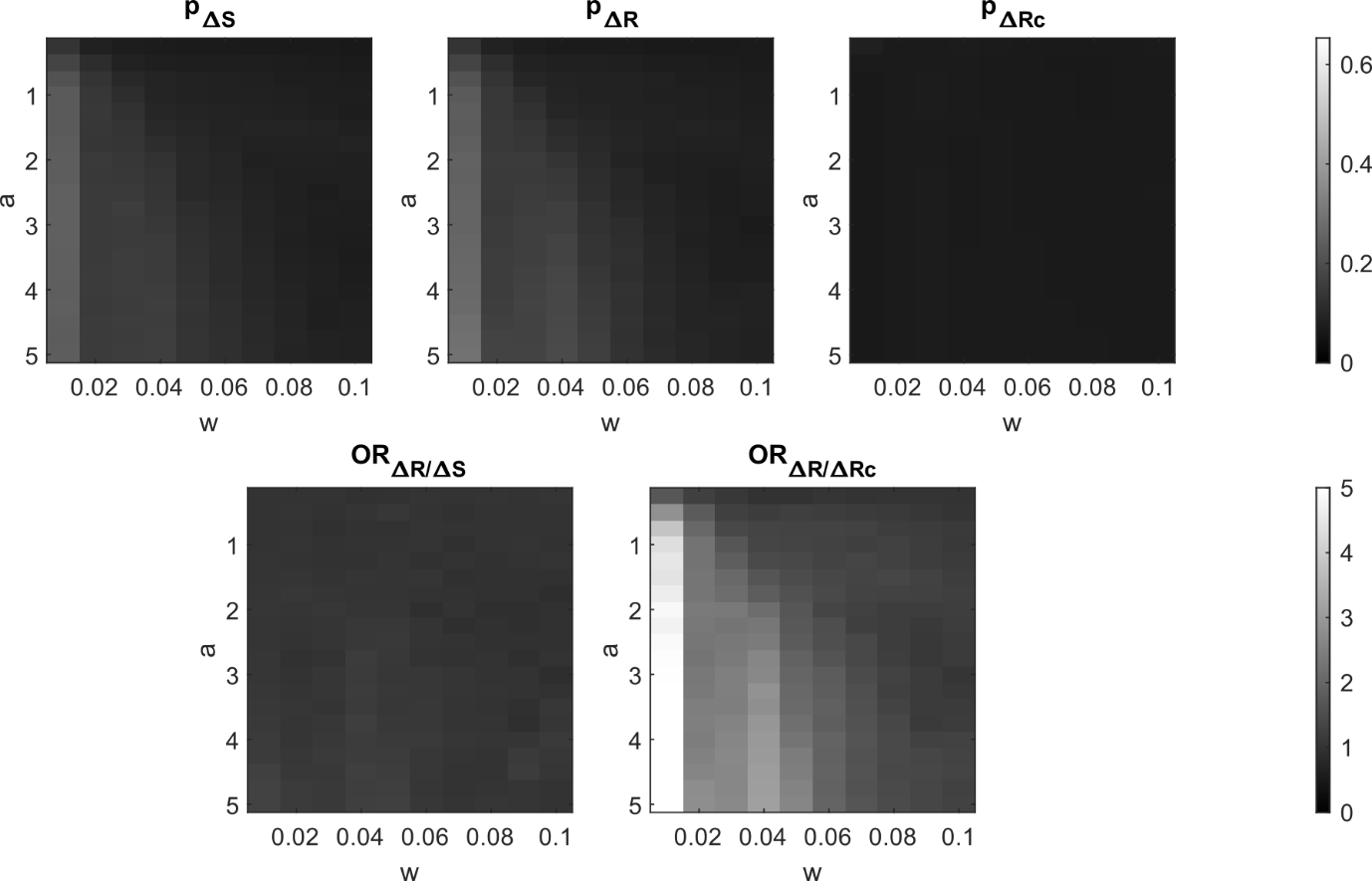


Figure 1S: A-C) Probabilities of spurious associations between current error and 3-back relative stimulus for different values of a and w coefficients in the 1-back generative models (eq. 3 and 4). A) Probabilities for the ∆S generator model. B) Probabilities for the ∆R generator model. C) Probabilities for the ∆R generator model after controlling for the 1-back response effect. D) Odds ratios of spurious associations for the ∆R generator model against those for the ∆S generator model. E) Odds ratios of spurious associations for the ∆R generator model before against after controlling for the 1-back response effect.


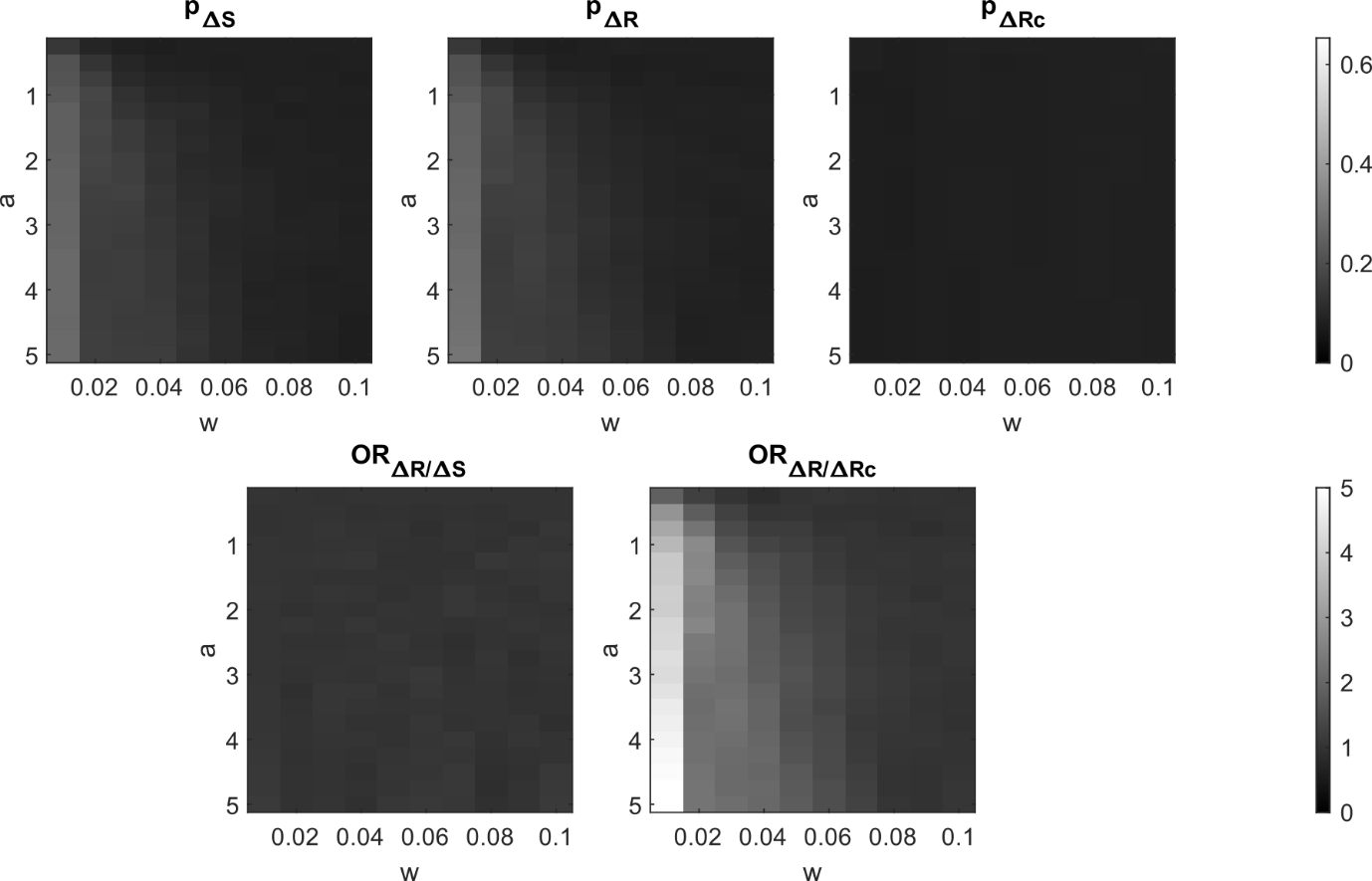


Figure 2S: A-C) Probabilities of spurious associations between current error and 4-back relative stimulus for different values of a and w coefficients in the 1-back generative models (eq. 3 and 4). A) Probabilities for the ∆S generator model. B) Probabilities for the ∆R generator model. C) Probabilities for the ∆R generator model after controlling for the 1-back response effect. D) Odds ratios of spurious associations for the ∆R generator model against those for the ∆S generator model. E) Odds ratios of spurious associations for the ∆R generator model before against after controlling for the 1-back response effect.


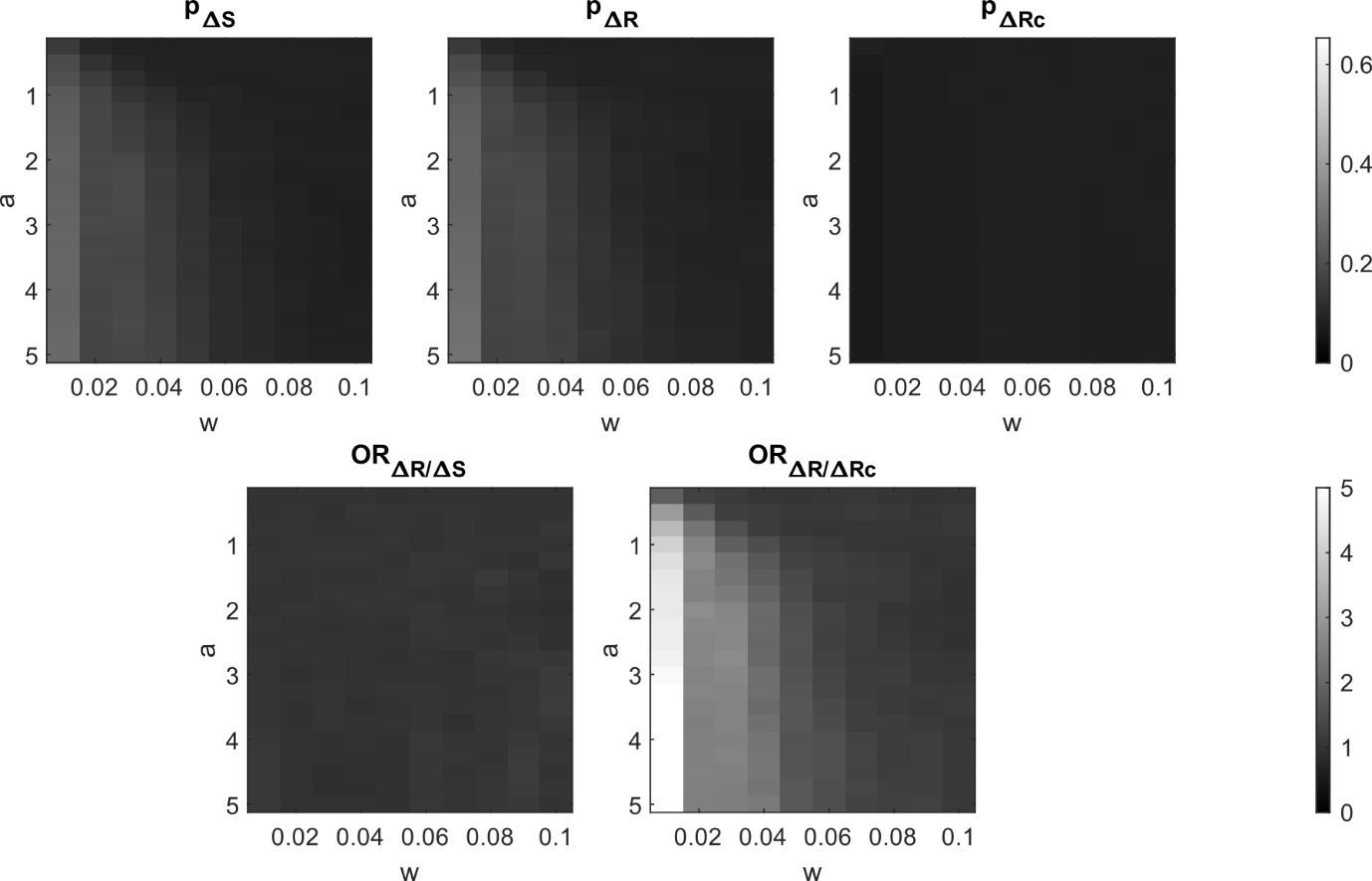


Figure 3S: A-C) Probabilities of spurious associations between current error and 5-back relative stimulus for different values of a and w coefficients in the 1-back generative models (eq. 3 and 4). A) Probabilities for the ∆S generator model. B) Probabilities for the ∆R generator model. C) Probabilities for the ∆R generator model after controlling for the 1-back response effect. D) Odds ratios of spurious associations for the ∆R generator model against those for the ∆S generator model. E) Odds ratios of spurious associations for the ∆R generator model before against after controlling for the 1-back response effect.
